# Clinical important improvement of chronic pain patients in randomized controlled trials and the DATA*PAIN* cohort

**DOI:** 10.1111/papr.13089

**Published:** 2021-11-29

**Authors:** Sophie Waardenburg, Nelleke de Meij, Brigitte Brouwer, Jan Van Zundert, Sander M.J. van Kuijk

**Affiliations:** ^1^ Department of Anesthesiology and Pain Medicine Maastricht University Medical Center+, MUMC+ Maastricht The Netherlands; ^2^ Department of Clinical Epidemiology and Medical Technology Assessment Maastricht University Medical Center+, MUMC+ Maastricht The Netherlands; ^3^ Department of Anesthesiology and Multidisciplinary Pain Centre Ziekenhuis Oost Limburg Genk/Lanaken Belgium

**Keywords:** chronic pain, clinically important improvement, cohort, Numeric Rating Scale, pain relief, randomized controlled trials

## Abstract

**Background:**

Change on the Numeric Rating Scale (NRS) is based on subjective pain experience, hampering the establishment of clinically important improvement. An anchor‐based method, the Patients’ Global Impression of Change (PGIC), is often added to determine whether a patient has improved. A two‐point change on the NRS has been shown to be equivalent to a moderate clinically important improvement in randomized controlled trials (RCT’s) on medication effects. We contemplated whether these findings could be reproduced in cohort and data and in non‐drug interventional RCT’s.

**Methods:**

The NRS change was quantified by subtracting the NRS of baseline from the NRS at 6‐month follow‐up. Categorization of success/nonsuccess was applied on the PGIC, and their average NRS raw changes were calculated. The Spearman correlation coefficient quantified the overall relationship, while the discriminative ability was explored through the receiver operating characteristic curve. Data were stratified on design, sex, and pain intensity at baseline. Besides, the cohort evaluated treatment status at follow‐up.

**Results:**

The records of 1661 patients were examined. Overall, the observed NRS change needed for moderate clinically important improvement was larger than the average two points. Yet, the changes in the cohort were smaller compared with the RCT’s. Moreover, it modified with pain intensity at baseline and treatment statuses indicated differences in mean clinical importance of −4.15 (2.70) when finalized at 6 months and −2.16 (2.48) when treatment was ongoing.

**Conclusion:**

The moderate clinically important improvement varied substantially, representing heterogeneity in pain relief and its relation to treatment success in chronic pain patients.

**Key points**

- Patients reported clinically important improvement at a larger pain relief than the average decrease of 2 points on the NRS.
- The amount of pain relief needed differed substantially between cohort and RCT patients.
- Pain severity at baseline modified the amount of pain relief needed for a clinically important improvement.

## INTRODUCTION

Many chronic pain patients do not obtain adequate or equivalent pain relief from existing interventions. Due to the highly subjective nature of pain, the meaningfulness of changes in pain is often difficult to interpret.^1^ Literature indicates a higher prevalence and average of pain intensity in women when compared to men.^2^, ^3^ Also, women seem to report greater functional limitations for the same pain intensity.^4^ Yet, it is rather unclear whether differences in meaningfulness of changes in pain are existent between sexes. The clinically important improvement in measurement scores is a critical consideration when evaluating treatment effect based on patient‐reported outcome measures (PROMs).^5^ The IMMPACT recommendations list the 11‐point Numeric Rating Scale (NRS) to quantify pain intensity and to summarize the subjective interpretation of the pain experienced. An essential step in clinical research is to determine the statistical significance and confidence intervals of the change in measurements scores within or between groups, as these reflect on the magnitude, variability of treatment effect, and sample size.^5^, ^6^ To determine the clinically important improvement, an anchor‐based method can be applied by relying on a global item completed by the patient, such as the Patients Global Impression of Change (PGIC). The PGIC does not primarily measure pain relief, but evaluates the overall improvement of the pain treatment. By anchoring these two measures, the relationship between pain relief and overall improvement can be examined from a patient point of view.^5^, ^6^, ^7^, ^8^

Several studies have quantified the clinically important improvement for several core domains in the chronic pain population.^1^, ^5^, ^6^, ^8^, ^9^, ^10^ The landmark paper of Farrar et al. assessed the clinical importance of pain relief. This was based only on data from randomized controlled trials (RCT’s) on the effect of pregabalin treatment.^6^ Ever since, an average change from baseline of two points or a 30% change on the NRS has often been regarded as a moderate clinically important improvement, that is equivalent to the categories “very much improved” and “much improved” on the PGIC.^6^

However, the chronic pain population is heterogeneous in pain relief and burden, and it is unclear whether these findings can be generalized toward patients included in interventional studies that analyze the effect of non‐drug treatments (eg, spinal cord stimulation and intradiscal injection), cohort data or subgroups of the chronic pain population. The aim of this study was to assess the generalizability of the aforementioned definition of the clinically important improvement on the NRS to non‐drug interventional RCT’s and heterogeneous cohort data. We expected to find a two‐point difference on average on the NRS as a moderate clinically important improvement in both interventional studies of non‐drug treatments and cohort data. However, we expected to find differences between subgroups of chronic pain populations, such as a larger difference than two points on average for women when compared to men. These results may contribute to the design of future studies, inform sample size calculations, and may set specific criteria for cohort studies. Such information will facilitate comparison of the results across studies and set the value for therapeutic meaningfulness in clinical practice.

## METHODS

For more than 20 years, the Department of Anaesthesiology and Pain Medicine of the Maastricht University Medical Center+in the Netherlands has routinely collected both the NRS and PGIC of chronic pain patients for both interventional studies and a cohort data. For this study, we used data of the RCT’s of van Eerd, et al.,^11^ Kallewaard et al.,^12^ Slangen, et al.,^13^ and Kemler et al.^14^ and cohort data of the DATA*PAIN* cohort, initiated in 2003 by the Comprehensive Multidisciplinary University Pain Center Maastricht.^2^ To perform this secondary analysis, approval was obtained by the medical ethical committee of the Maastricht University (METC approval number: 2020‐2391). All studies included followed the recommendations of the IMMPACT guidelines on core outcomes for an adequate evaluation of the treatment efficacy and effectiveness.^5^, ^15^ For this secondary analysis, patients were included if they were 18 years or older, had been experiencing pain for more than 3 months, and had completed both the NRS and the PGIC at 6‐month follow‐up.

### Measurements

The 11‐point NRS was used to quantify pain intensity ranging from zero (no pain) to ten (the most pain imaginable).^16^ In the RCT’s, the average momentary NRS was computed from a 4‐day diary at baseline and 6‐month follow‐up. In the cohort, the average NRS of the past week was collected at both measurement moments using a single item.

The PGIC was used to collect the status of the patient's global impression of change on a 7‐point Likert scale ranging from “very much improved” to “very much worse”. In addition, a dichotomous PGIC score was computed in which “very much improved” and “much improved” indicated a successful treatment outcome and “minimally improved,” “no change,” “minimally worse,” “much worse,” and”very much worse,” a non‐successful outcome. The PGIC was used as an anchor‐based criterion to distinguish between successful and non‐successful treatment at follow‐up.^5^

#### Stratification

Because the literature suggests analyzing both sexes separately as there may be different values for clinically important improvement on treatment outcome,^5^ we stratified on sex in addition to study design (ie, cohort and RCT). Moreover, baseline NRS scores were cutoff into 3 different pain categories: mild, with a pain intensity of 0–5 on the NRS; modest, with a 5–7 on the NRS; and severe, with a 7–10 on the NRS.^17^ In the DATA*PAIN* cohort, patients could have been treated for more than 6 months (the follow‐up time used to compute change from baseline) due to receiving more than one treatment or elongation of a specific treatment. Therefore, the cohort was stratified on treatment status at follow‐up; completed; or ongoing.

### Statistical analysis

Study‐level characteristics (age, sex, and NRS scores) were described as means and standard deviations (SD) or percentages. The NRS change was quantified by subtracting the baseline NRS from the follow‐up NRS and described as a mean difference with 95% confidence interval (CI). To test within‐group changes, the paired sample *t*‐test was applied.

Average raw and relative changes in the NRS were calculated for each of the 7 outcome categories of the PGIC, and the Spearman correlation coefficient was calculated to quantify this relationship. Furthermore on the PGIC, patients were classified into treatment success or nonsuccess and the respective NRS changes were calculated. To assess the discriminative ability of the NRS for treatment success, the area under the curve receiver operating characteristic curve was computed, or AUC, with 95% CI. The AUC can range between 0.5 (no discriminative ability) and 1.0 (perfect discriminative ability).

Subsequently, the data were stratified and analyses were repeated for the study designs (RCT and cohort), sex categories, baseline NRS categories, and treatment status in the cohort data. The statistical analyses were executed in R, a language for statistical computing, version 3.6.1.

## RESULTS

Table 1 summarizes the study characteristics and baseline variables of the cohort and RCT’s. In case of the latter, the variables were presented for each separately and all RCT’s combined. The results of the Spearman correlations and ROC curve analyses are summarized in Table 2. In total, the records of 1661 chronic pain patients were examined. In this study, an average raw NRS change of −3.58 (SD: 1.89) was associated with a clinically important improvement as defined on the PGIC. This average was much higher than expected based on the two points or more raw change found in the literature. Moreover, the NRS change required in the cohort data differed from the RCT’s.

**TABLE 1:** Study and patient characteristics

Study name	No. pts. (%)	Age mean (SD)	Women in %	Baseline pain mean (SD)	Follow‐up pain mean (SD)	Mean difference (CI)	*p* Value^a^
DATA*PAIN* cohort	1424 (100)	60.24 (13.24)	55.66	7.21 (1.67)	5.92 (2.40)	−1.30 (−1.42, −1.17)	<0.001
All RCTs	237 (100)	49.12 (14.43)	55.60	6.80 (1.66)	4.86 (2.79)	−1.94 (−2.28, −1.60)	0.006^b^
RCT: PDP^c^	32 (13.5)	57.59 (10.67)	31.25	6.87 (1.69)	5.15 (2.74)	−1.72 (−0.80, −2.65)	<0.001
RCT: IMBI^d^	76 (32.08)	41.45 (9.74)	69.74	6.58 (0.99)	5.14 (2.56)	−1.44 (−0.89, −1.99)	<0.001
RCT: RFD^e^	75 (31.65)	60.45 (11.15)	44	6.96 (1.08)	4.15 (2.78)	−2.81 (−2.19, −3.44)	<0.001
RCT: ESES^f^	54 (22.78)	38.63 (10.90)	68.52	6.84 (1.41)	5.27 (3.01)	−1.57 (−0.84, −2.29)	<0.001
DATA*PAIN* WOMEN	793 (55.69)	59.10 (14.07)	100	7.32 (1.64)	6.02 (2.43)	−1.30 (−1.47, −1.14)	<0.001
DATA*PAIN* MEN	631 (44.31)	61.68 (12.21)	0	7.09 (1.70)	5.79 (2.37)	−1.29 (−1.47, −1.11)	<0.001
RCT WOMEN	133 (56.12)	43.35 (14.36)	100	6.79 (1.25)	4.97 (2.75)	−1.82 (−2.27, −1.37)	<0.001
RCT MEN	104 (43.78)	52.39 (14.36)	0	6.81 (1.22)	4.72 (2.85)	−2.10 (−2.62, −1.58)	<0.001
DATA*PAIN* TX finished	416 (34.44)	61.19 (13.04)	52.42	7.03 (1.03)	4.90 (2.71)	−2.13 (−2.39, −1.86)	<0.001
DATA*PAIN* TX ongoing	520 (43.05)	58.96 (13.68)	58.05	7.36 (1.59)	6.61 (1.94)	−0.75 (−0.91, −0.59)	<0.001
DATA*PAIN* Mild NRS	107 (7.51)	60.93 (13.70)	49.53	3.30 (0.92)	394 (2.40)	0.65 (0.16, 1.13)	0.009
DATA*PAIN* Modest NRS	253 (17.77)	60.21 (12.82)	53.75	5.57 (0.50)	4.99 (2.24)	−0.57 (−0.85, −0.30)	<0.001
DATA*PAIN* Severe NRS	1064 (74.72)	60.18 (13.43)	56.77	8.00 (0.89)	6.34 (2.28)	−1.66 (−1.79, −1.53)	<0.001
RCT’s Mild NRS	14 (5.91)	47.29 (14.98)	57.14	4.20 (0.44)	2.59 (1.84)	−1.60 (−2.75, −0.45)	0.009
RCT’s Modest NRS	91 (38.40)	47.23 (14.84)	58.24	5.90 (0.53)	4.34 (2.63)	−1.57 (−2.11, −1.03)	<0.001
RCT’s Severe NRS	132 (55.70)	50.4 (13.87)	54.55%	7.69 (0.68)	5.46 (2.79)	−2.23 (−2.70, −1.77)	<0.001

Abbreviations: CCFJP, chronic cervical facet joint pain; CDLBP, chronic discogenic low back pain; DPN, diabetic peripheral neuropathy; IMBI, intradiscal methylene blue injection; PDP, painful diabetic poly‐neuropathy; pts., patients; RFD, radiofrequency denervation; RSD, reflex sympathetic dystrophy; SCS, spinal cord stimulation; TX finished, patient treatment finished at 6‐month follow‐up; TX ongoing, patient treatment ongoing at 6‐month follow‐up.

^a^ Paired *t*‐test.

^b^ ANOVA.

^c^ Study; PDP, Diagnosis; DPN, Intervention; SCS.

^d^ Study; IMBI, Diagnosis; CDLBP, Intervention; IMBI.

^e^ Study; RFD, Diagnosis; CCFJP, Intervention; RFD.

^f^ Study; ESES, Diagnosis; RSD, Intervention; SCS.

**TABLE 2:** Roc curve analyses and correlations

Strata	Area under the ROC curve (CI)	Percent agreement	Chi‐square test *p* value	Spearman correlation = rho, *p* value
Study design				
Cohort	0.79 (0.76, 0.82)	72.6	<0.001	0.46, <0.001
RCT’s	0.93 (0.76, 0.85)	84	<0.001	0.72, <0.001
SEX in cohort				
Women	0.79 (0.73, 0.82)	19.4	<0.001	0.46, <0.001
Men	0.80 (0.76, 0.84)	18.5	<0.001	0.43, <0.001
Sex in RCT’s				
Women	0.95 (0.91, 0.98)	13.8	<0.001	0.79, <0.001
Men	0.91 (0.86, 0.97)	18.3	<0.001	0.75, <0.001
Duration pain treatment in cohort				
<6 months	0.81 (0.76, 0.85)	22.6	<0.001	0.58, <0.001
>6 months	0.69 (0.63, 0.77)	16.9	<0.001	0.31, <0.001
NRS baseline scores—Cohort				
Mild	0.75 (0.44, 0.86)	21.5	0.006	0.50, <0.001
Modest	0.78 (0.71, 0.85)	19.8	<0.001	0.48, <0.001
Severe	0.82 (0.79, 0.83)	18.6	<0.001	0.48, <0.001
NRS baseline scores—RCT’s				
Mild	0.86 (0.61, 1)	21.4	0.11	0.63, 0.01
Modest	0.96 (0.93, 1)	12.1	<0.001	0.77, <0.001
Severe	0.92 (0.88, 1)	17.4	<0.001	0.71, <0.001

### Cohort versus RCT’s

**FIGURE 1:**
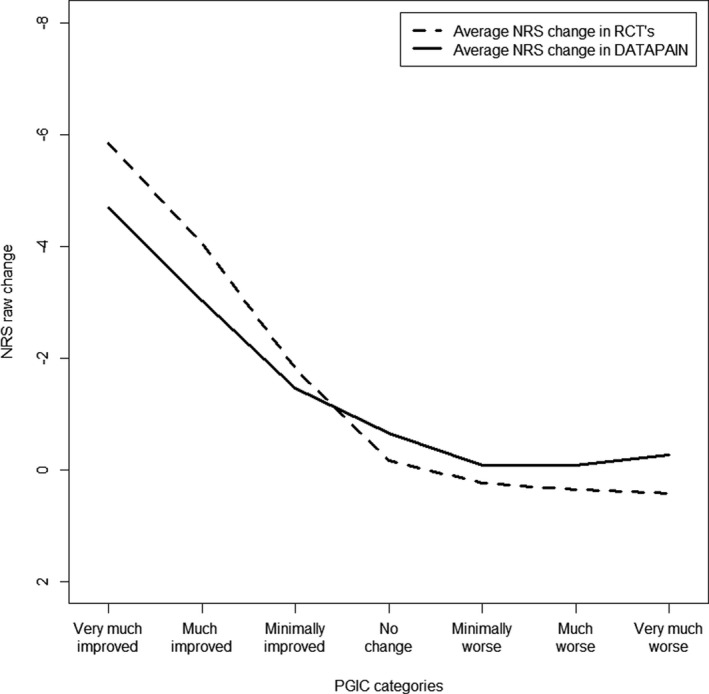
Average NRS change score for the PGIC categories of the cohort and RCT’s

When comparing the cohort data with the RCT’s, the average NRS changes differed for each of the PGIC categories (Figure 1). Similarly, the success/nonsuccess PGIC differed in average raw NRS changes, with averages of −3.33 (SD: 2.72) for the cohort and −4.56 (SD: 1.85) for the RCT’s (*p* < 0.001). Hence, cohort participants reported treatment success at smaller changes in pain relief, on average. Moreover, the percentage of treatment success diverged considerably between the cohort with 316 (22.19%) patients and 81 (34.18%) in the RCT’s (*p* < 0.001).

### Stratification on sex

**FIGURE 2:**
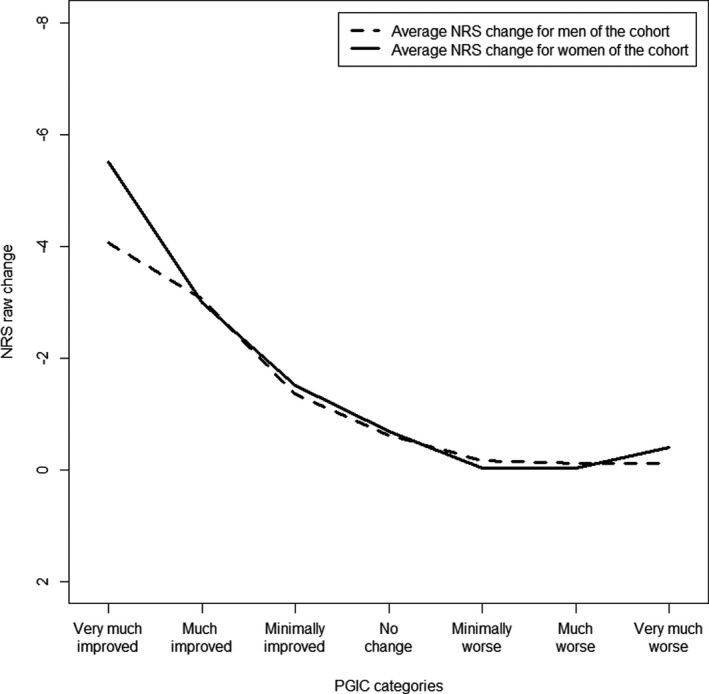
Average NRS change score for the PGIC category in men and women of the cohort

**FIGURE 3:**
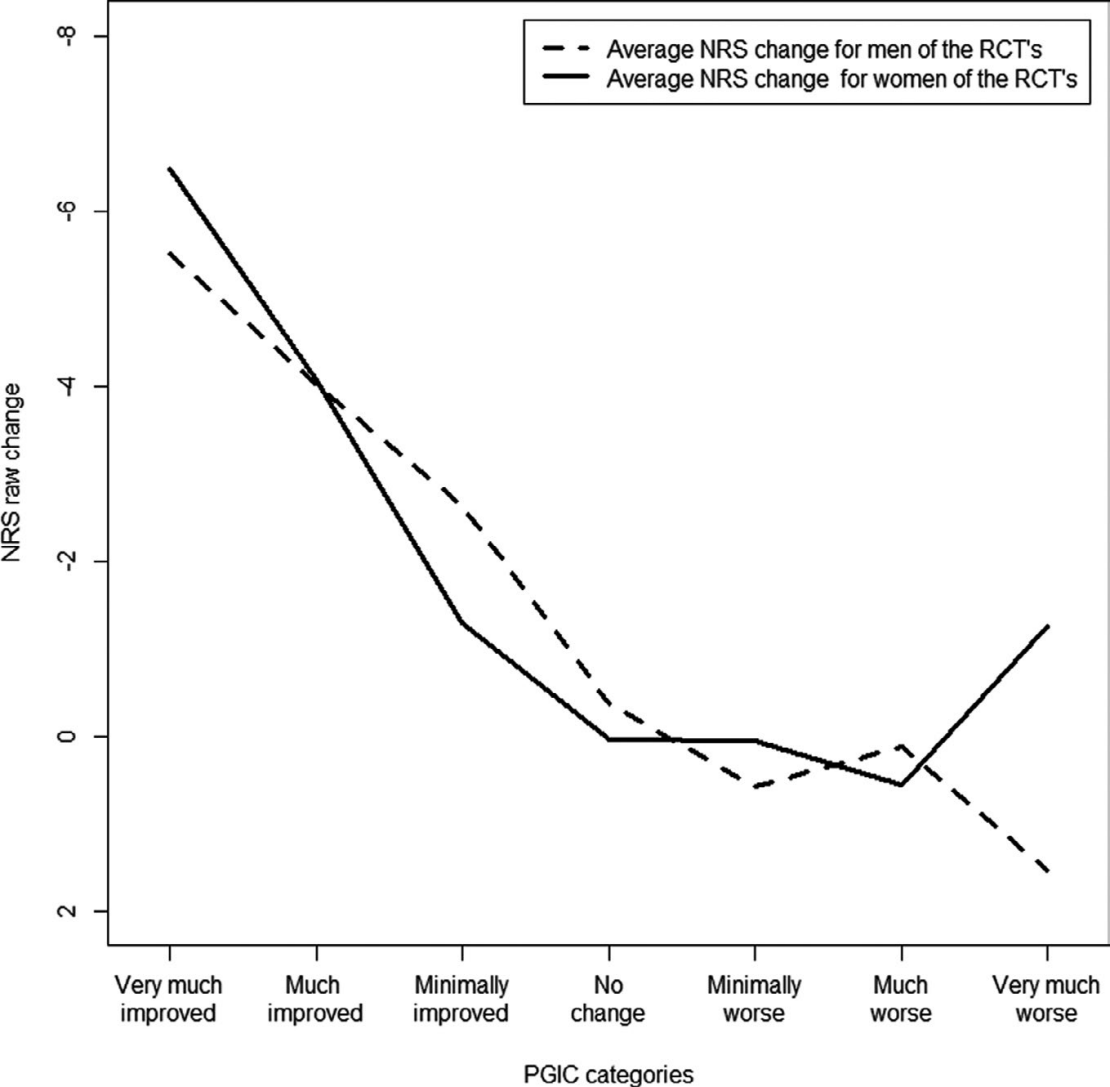
Average NRS change score for the PGIC category in men and women of the RCT’s

In both the cohort and RCT’s, the stratification on sex resulted in different mean values of clinically important improvement in the”very much improved” categories of the PGIC (Figures 2 and 3). Women indicated to need, on average, one point more in NRS change to label their improvement as “very much improved.” Nonetheless, the average NRS changes for treatment success did not differ between the sexes, in the DATA*PAIN* cohort; −3.38 (SD: 2.82) for women and −3.28 (SD: 2.61) for men, nor in the RCT’s; −4.49 (SD: 1.84) for women and −4.65 (SD: 1.88) for men.

### DATA*PAIN* cohort; stratification on treatment status

**FIGURE 4:**
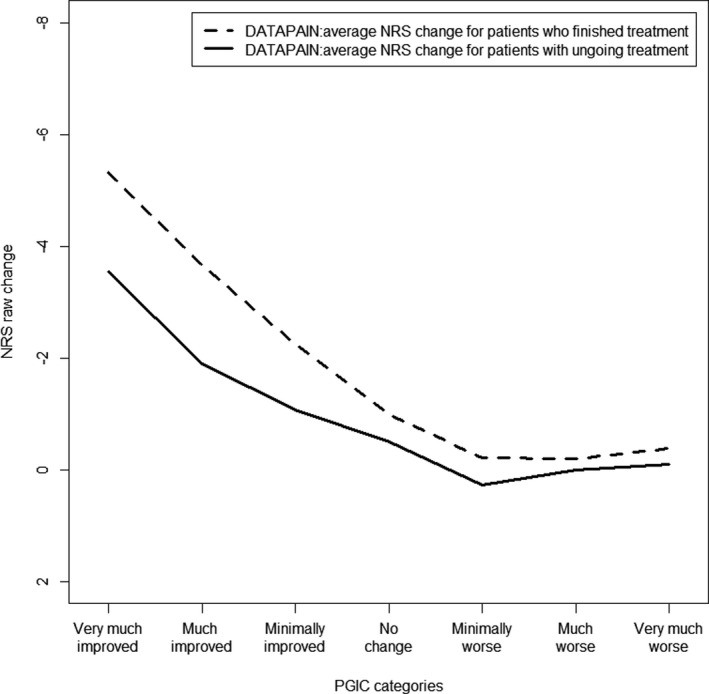
Average NRS change score for the PGIC categories in treatment status of the cohort

In the cohort, the treatment duration resulted in considerably different NRS changes between baseline and follow‐up. The average raw NRS change, for the completed treatment group, was −2.13 (95% CI: −2.39, −1.86) and −0.79 (95% CI: −0.95, −0.63) for the ongoing treatment group (*p* < 0.001) (Figure 4). Besides, the percentage of treatment success differed significantly, for those who had completed treatment (151 patients, 36.3%) and those ongoing in treatment (72 patients, 13.85%), *p* < 0.001. To report clinically important improvement, an average NRS change of −4.15 (SD: 2.70) was needed for the completed group and a −2.16 (SD: 2.48) for the ongoing group. Thus, the ongoing treatment group reported to experience treatment success at a much lower average NRS change compared with those with completed treatment.

### Stratification on NRS baseline score

**FIGURE 5:**
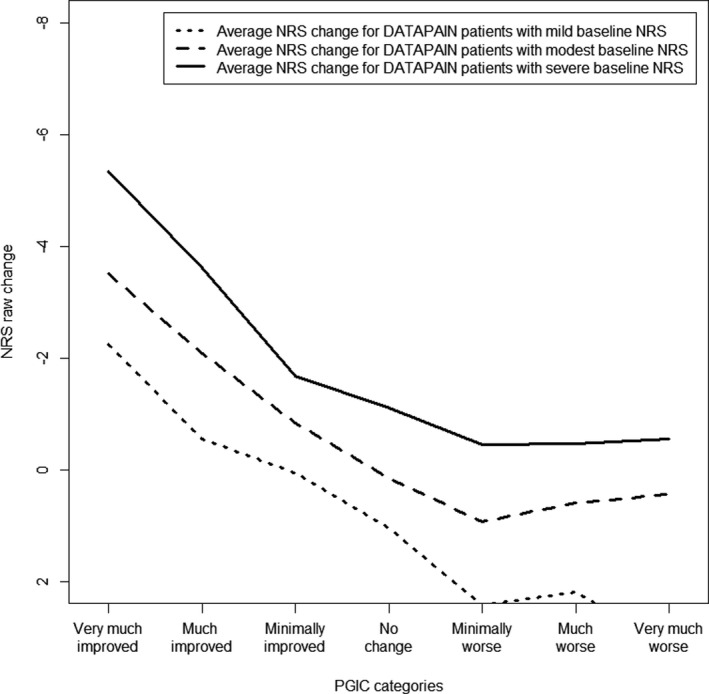
Average NRS change score for the PGIC categories stratified on NRS baseline in the cohort

**FIGURE 6:**
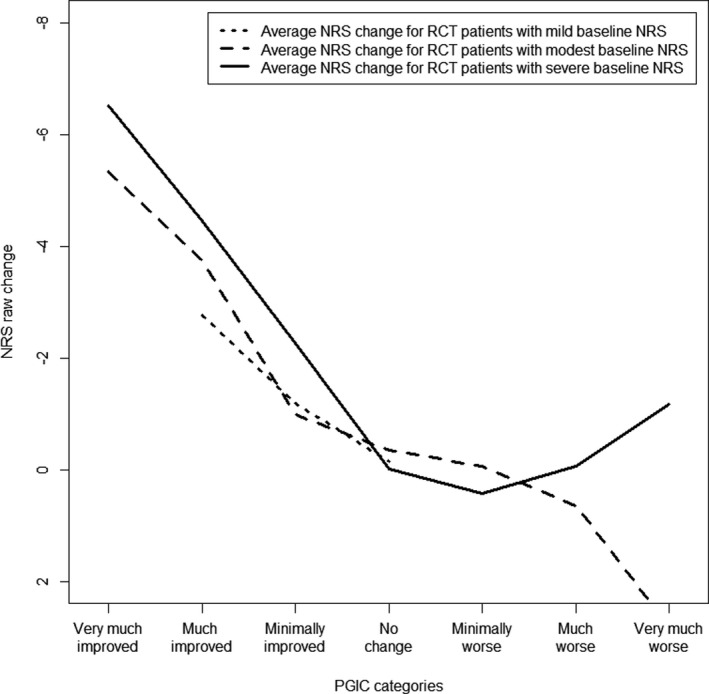
Average NRS change score for the PGIC categories stratified on NRS baseline in the RCT’s

Differences in pain severity at baseline were more prominent in the cohort data than in the RCT’s (Figures 5 and 6). A clinically important improvement was observed at larger average NRS changes when patients were part of the severe NRS groups: −3.95 (SD:2.62) for the cohort and −4.90 (SD:1.99) for the RCT’s, when compared to the modest NRS group: −2.25 (SD:2.33) for the cohort and −4.45 (SD:1.52) for the RCT’s, and mild NRS groups: −1.04 (SD:2.35) for the cohort and −2.76 (SD:0.96) for the RCT’s. Suggesting that, independently of study design, an expectation of the amount of pain reduction may be present that depends on the severity of pain at baseline.

## DISCUSSION

The main objective of this study was to assess whether an average decrease of two points on the NRS was found to be clinically important, in chronic pain patients who participated in a large observational cohort and in patients included in RCT’s of non‐pharmaceutical treatments. In this study, patients reported clinically important improvement at a larger pain relief than the average decrease of two points on the NRS. The amount of pain relief needed differed substantially between study designs. In both cohort and RCT’s, pain severity at baseline modified the amount of pain relief needed for a clinically important improvement. Furthermore within the cohort, considerable differences were found in treatment status.

In the RCT’s, strict inclusion criteria were applied before administering a single intervention, while personalized care was provided to every patient in the cohort, as these patients received care of a multidisciplinary pain team. Therefore, cohort patients may have had more than one intervention or have been treated intermittently, leading to patients in treatment at 6‐month follow‐up. We believed that this had an influence on the NRS change and the value given to the clinical importance of the improvement. Stratification revealed that the change in pain relief was reduced significantly when observing clinically important improvement for those still being treated. Illustrating that patients in treatment at 6 months were satisfied with their treatment progress at a much lower rate in pain relief. Notwithstanding, the association between the NRS change and clinically important improvement decreased substantially in the AUC, specifying that for these patients other underlying factors may play an important role when answering the PGIC. Therefore, the need for further investigation on factors that contribute in answering the PGIC is warranted.

The stratification on baseline NRS indicated that NRS change is non‐uniform across these groups.^6^, ^8^ Care should be taken in the comparison with patients that initiate at different pain intensities at baseline. This may be due to certain expectancy of improvement, hence further research on this specific topic may clarify the motive. The differences in the RCT’s were not all significant, plausibly due to the low amount of patients included in the mild NRS baseline group, as per inclusion criteria, and hence, low statistical power may have caused us to miss differences.

Stratification on sex revealed no differences at the two‐point raw change representing the two successful categories of the PGIC. Furthermore, a patient characteristic that may play a role as well is age. We observed on the cross‐sectional level in our tertiary pain population patients of older age have a lower average pain intensity at presentation.^3^ Yet, a longitudinal study on birth cohorts indicate that there is a positive relationship between age and pain intensity within patients over time.^18^ Socio‐demographic variables that have a negative association with pain are education, employment, and wealth.^3^, ^18^ Yet, the question remains if these factors have influence on the outcome of the PGIC and are recommended to be further analyzed in future studies.

The results of this study show different outcomes when compared to the results of Farrar et al; this may be due to the type of study design or intervention under analysis. Expectations on the effect of treatment on pain decrease seems dependent on many characteristics of patients and the setting they were recruited. It seems that patients are not satisfied with just a two‐point difference. An option would be to adjust the definition of clinical important improvement. Nevertheless, in clinical practice, only a low rate of the pain population obtains a successful intervention. By increasing the clinical importance, to for example a three‐point raw change, this will have a negative impact on our view of their efficacy and it may have implications for future study design, such as necessary sample sizes. Another option could be to analyze the importance and sensitivity of composite scores or quality of life scores as pain outcome measures.

In general, the Spearman correlation coefficient suggested that pain change is an important component measured by the PGIC, yet the lack of strong correlations shows that pain relief cannot explain treatment success in full. Moreover, relative changes were omitted in the results as they were interchangeable with the raw changes, making the interpretation easier when only the latter were included. This is due to the high baseline pain scores of the patients we have at the tertiary pain clinic, making the relationship between the raw change and PGIC as stable as the percent changes and PGIC. The questionnaires applied in both methods were validated for the chronic pain population and recommended by the IMMPACT guidelines, reducing the probability of errors in data collection. In both methods, application of stratification controlled for confounding.^19^ Overall, consistency in association and performance was found between the change in pain intensity and meaningfulness of the PGIC, regardless of the treatment patients received, sex, treatment status, or baseline pain intensity, suggesting a high external validity toward the diverse chronic pain population. Consequently, the application of the results may provide indications on clinically important improvement, contribute in calculating sample size and number needed to treat in future studies, not only in randomized controlled trials, but also in cohort data for chronic pain patients referred to tertiary pain clinics.

In conclusion, the change in NRS scores associated with clinically important improvement was larger than is stipulated in the literature and the amount of pain relief needed was substantially larger in RCT’s than in cohort data. Stratification on study design and sex showed the presence of heterogeneity in the pain relief and its significance in relation to treatment success, calling for caution in the interpretations as is it may be dependent on study design, NRS at baseline, or treatment status.

## CONFLICT OF INTEREST

The authors have no conflicts of interest to declare.

## AUTHORS’ CONTRIBUTION

All authors have contributed to the conception of the study design. Data collection and statistical analyses were carried out by the first author, with critical discussion and revision from all authors. All authors have read and approved the manuscript.

## Data Availability

Data are available upon reasonable request.
